# Effects of exercise on glycolipid metabolism in adolescents with overweight and obesity: a systematic review and meta-analysis of 26 randomized controlled trials

**DOI:** 10.7717/peerj.19365

**Published:** 2025-04-23

**Authors:** Xinyu Fan, Weihao Sun, Song Gu

**Affiliations:** China Volleyball College, Beijing Sport University, Beijing, China

**Keywords:** Glycolipid metabolism, Overweight, Obese, Adolescents, Exercise, Systematic reviews

## Abstract

**Objective:**

The aim of this meta-analysis was to investigate the effect of exercise intervention on glycolipid metabolism in overweight and obese adolescents.

**Methods:**

A systematic review and meta-analysis of randomized trials were conducted. The review adhered to the Preferred Reporting Items for Systematic Reviews and Meta-Analyses (PRISMA) guidelines and was registered (ID: CRD42024623686). Electronic searches were performed using the following databases: Web of Science, PubMed, Scopus, Cochrane and Embase. Randomized controlled trials of exercise interventions were included. Data on fasting blood glucose (FBG), fasting insulin (FINS), total cholesterol (TC), triglycerides (TG), low-density lipoprotein cholesterol (LDL-C) and high-density lipoprotein cholesterol (HDL-C) before and after exercise interventions were extracted for overweight and obese adolescents. Risk of bias was assessed using the Cochrane risk of bias tool. The Grading of Recommendations, Assessment, Development, and Evaluation (GRADE) tool was used to evaluate the quality of the evidence. Standardized mean differences (SMDs) were calculated to compare differences between exercise and conventional control groups. Subgroup analyses were performed to assess whether effects differed by exercise type, intervention duration, supervision, and intervention frequency.

**Results:**

A total of 984 participants (576 in experimental groups and 408 in control groups) were included across 26 studies. The analysis revealed that exercise interventions significantly improved key metabolic parameters: FBG (SMD: −0.42 95% CI [−0.73 to −0.12]), FINS (SMD: −0.81 95% CI [−1.13 to −0.49]), TC (SMD: −0.18 95% CI [−0.34 to −0.01]), TG (SMD: −0.46 95% CI [−0.56 to −0.25]), LDL-C (SMD: −0.28 95% CI [−0.44 to −0.12]), and HDL-C (SMD: 0.26 95% CI [0.11–0.40]). Subgroup analysis indicated that exercise type, supervision and intervention frequency influenced the effectiveness.

**Conclusion:**

The analysis suggests that exercise interventions improve glycolipid metabolism in adolescents with overweight and obesity. Continuous endurance training demonstrated greater efficacy in improving blood glucose parameters, whereas hybrid-type exercise showed advantages in improving lipid metabolism. Engaging in three supervised training sessions weekly may be the optimal approach to enhance glycolipid metabolism in obese adolescents. These findings provide evidence for clinicians and healthcare professionals (*e.g*., exercise physiologists, physical therapists) to guide exercise prescriptions for obese adolescents, thereby preventing worsening metabolic imbalances.

## Introduction

Adolescent obesity has emerged as a critical global public health challenge, exerting widespread impacts on socio-economic development, including physical growth, mental health, social adaptation, fertility, workforce productivity, and disease burden in children and adolescents. According to the World Health Organization (WHO), the prevalence of overweight and obesity among children and adolescents has risen eightfold over the past four decades (World Health Organization, 2023), reaching epidemic levels globally (Bradwisch et al., 2020). Current estimates indicate that 14.8% of children and adolescents are obese, while 22.2% are overweight, translating to approximately one in five individuals affected (Zhang et al., 2024). This escalating trend necessitates urgent attention. Overweight and obesity have been shown to detrimentally affect adolescents’ physical and mental health, increasing susceptibility to hypertension, respiratory disorders, certain cancers, and cardiovascular diseases (CVD) (McKinsey Global Institute, 2014), with a strong association with glucose and lipid metabolism disorders (GLMD) (Huang, Wu & Deng, 2022). For instance, a study of individuals aged 5–19 years revealed that overweight and obese adolescents, particularly those classified as obese, exhibit elevated fasting blood glucose (FBG) and fasting insulin (FINS) levels (Chen et al., 2022). Furthermore, obesity is closely linked to dyslipidemia: the prevalence of hyperlipidemia in obese individuals is significantly higher than in normal-weight populations (Zhou et al., 2025), with consistent positive correlations observed between obesity and total cholesterol (TC), triglycerides (TG), and low-density lipoprotein cholesterol (LDL-C) and a negative correlation with high-density lipoprotein cholesterol (HDL-C) (Al-mhanna et al., 2024). Notably, a longitudinal study reported a marked increase in dyslipidemia cases among individuals with a body mass index (BMI) ≥30 kg/m^2^, identifying obesity as the primary risk factor for abnormal lipid profiles (Tang et al., 2022).

Obesity is characterized by excessive adipose accumulation and chronic energy surplus, resulting from genetic predisposition and behavioral factors. Adipose tissue, a metabolically active endocrine organ, secretes inflammatory cytokines that disrupt systemic homeostasis (Gregor & Hotamisligil, 2011), directly impairing glycolipid metabolism. Glycolipid metabolism, essential for energy production and physiological function, when dysregulated, serves as a key risk factor for atherosclerotic diseases, insulin resistance, diabetes, and vascular or neurological complications such as stroke (Tang et al., 2021), as well as organ damage (*e.g*., heart, liver, kidneys) (Asrih & Jornayvaz, 2015).

The rapid rise in glycolipid metabolic disorders, driven by high-fat diets and sedentary lifestyles, poses a severe threat to global health (Han, Abdul Malik & Hashim, 2024). Current pharmacological therapies for GLMD remain suboptimal, as synthetic drugs primarily target isolated metabolic markers without addressing the underlying problem of “energy accumulation” as a whole (Agudelo et al., 2018), while also carrying risks of adverse effects and high costs (Tak & Lee, 2021). In contrast, exercise therapy has demonstrated efficacy in mitigating metabolic dysfunction. The 2020 WHO Guidelines on Physical Activity for Children and Adolescents recommend ≥60 minutes of daily moderate-intensity aerobic exercise to improve cardiometabolic health and reduce sedentary behavior (Chaput et al., 2020). Exercise interventions not only reduce adiposity and enhance fitness in overweight adolescents but also improve body composition, glucose and lipid profiles, blood pressure, and cardiorespiratory fitness (O’Donoghue et al., 2021), thereby lowering the risk of type 2 diabetes and CVD (Bülbül, 2020) without adverse effects (LaMonte et al., 2018). Thus, exercise is pivotal for weight management, metabolic health improvement, and disease prevention in obese populations. However, the efficacy of exercise in ameliorating glycolipid metabolism in adolescents remains inconclusive, owing to limited high-quality randomized controlled trials (RCTs) and meta-analyses. Existing evidence is conflicting: while some studies suggest that exercise improves GLMD (Batrakoulis et al., 2022; LaMonte et al., 2018) and cardiometabolic health (Haring et al., 2023), others report minimal benefits on glycemic and lipid markers when exercise is implemented independently (Hadgraft et al., 2021; Khalafi et al., 2023; Pozuelo-Carrascosa et al., 2018b). Notably, many findings derive from adult or clinical populations, limiting their generalizability to adolescents.

This systematic review and meta-analysis aims to: (1) evaluate the effects of exercise interventions on glycolipid metabolism outcomes (FBG, FINS, TC, TG, LDL-C, HDL-C) in obese adolescents; (2) elucidate exercise’s preventive and therapeutic potential against GLMD; (3) assess the influence of exercise modality, supervision, duration, and frequency on efficacy; (4) identify optimal exercise regimens for GLMD prevention in this population.

## Methods

### Registration

This systematic review and meta-analysis adhered to the Preferred Reporting Items for Systematic Reviews and Meta-Analyses (PRISMA) guidelines (Higgins et al., 2003) and was registered in the PROSPERO database (Registration ID: CRD42024623686).

### Literature search strategy

A comprehensive search was performed across five databases: Web of Science, PubMed, Scopus, Cochrane Library, and Embase, combining Medical Subject Headings (MeSH) terms with free-text keywords related to “glycolipid metabolism”, “exercise”, and “obese adolescents”. The search covered records from database inception to November 2024. Non-peer-reviewed publications (*e.g*., books, conference abstracts, letters) and non-English articles were excluded during the search. The full search strategy (including Boolean operators and term combinations) is provided in Table S1.

### Selection criteria

Eligibility was determined using the PICOS framework (Participants, Interventions, Controls, Outcomes, Study Design) (Sherrington et al., 2000): (1) Participants: Overweight or obese adolescents aged 7–19 years, defined by WHO criteria as BMI ≥24 kg/m^2^ and <30 kg/m^2^ (overweight) or BMI ≥30 kg/m^2^ (obesity) (World Health Organization, 2020). (2) Intervention: Structured exercise programs lasting ≥4 weeks, defined as “planned, organized, and repetitive physical activity aimed at improving fitness or health” (Garber et al., 2011). Exercise modalities included: continuous endurance training (CET), resistance training (RT), combined training (CT), interval training (INT), and hybrid exercise (HYB) (see Table S2 for definitions). (3) Control: Non-exercising groups (*e.g*., usual care, sedentary controls). (4) Outcomes: Glycemic markers (FBG, FINS) and lipid profiles (TC, TG, LDL-C, HDL-C). (5) Study design: Randomized controlled trials (RCTs).

Exclusion criteria: Non-English articles; duplicates; reviews, editorials, or animal studies; studies with unavailable full texts; multi-component interventions (*e.g*., diet + exercise) where exercise effects could not be isolated; studies lacking glycolipid metabolism outcomes.

### Study selection

Two independent reviewers (X.F., W.S.) screened titles/abstracts and assessed full texts against inclusion criteria. Discrepancies were resolved by a third reviewer (S.G.). Manual searches of reference lists were conducted to identify additional eligible studies.

### Data extraction

Two reviewers (X.F., W.S.) independently extracted data into a standardized Excel template, including study characteristics (authors, year, design), participant demographics (sample size, sex, age, BMI), intervention details (exercise type, intensity, duration, frequency, session length and supervised status, adherence rate) and relevant outcomes (pre- and post-intervention means and standard deviations for FBG, FINS, TC, TG, LDL-C, HDL-C). Disagreements were adjudicated by S.G.

### Risk of bias assessment and quality assessment

Risk of bias was evaluated using the Cochrane Risk of Bias Tool (Higgins et al., 2011). The Cochrane Collaboration tool appraised specific items in the domain of bias (unclear, high or low) based on decision-making processes, encompassing selection bias (randomized sequence generation and allocation concealment), reporting bias (selective reporting), performance bias (blinding of personnel and participants), detection bias (blinded outcome assessment), attrition bias (incomplete data) and other biases (other sources of bias) were reported. Due to the inherent challenges of blinding participants in exercise trials, performance bias was not assessed. Two reviewers (X.F., W.S.) independently rated each domain as low, high, or unclear risk. Discrepancies were resolved through consensus.

Evidence quality was graded using the Grading of Recommendations, Assessment, Development, and Evaluation (GRADE) framework (Guyatt et al., 2011). The quality of the evidence for each outcome was classified as high, moderate, low or very low depending on the study design. Subsequently, due to the subjective nature of decision-making, the following criteria were used to downgrade or upgrade the quality of the evidence: (1) risk of bias (downgrade one level when <75% of the included studies are at low risk of bias); (2) inconsistency (downgrade one level when I^2^ > 50%); (3) indirect evidence (*i.e*., indirect populations, interventions, comparisons, or outcomes); (4) imprecision as indicated by wide confidence intervals; and (5) publication bias which downgrades the quality of the evidence (Guyatt et al., 2011; Schünemann et al., 2019).

### Statistical analyses

The extracted data were analyzed using a combination of narrative and quantitative synthesis. Narrative synthesis entails the summary of basic information about the included studies and the risk of bias. Quantitative synthesis necessitates the execution of either random or fixed-effect meta-analysis of the mean and standard deviation (SD) of alterations in pertinent outcome indicators in post-intervention studies, in conjunction with the sample size (N). We performed separate meta-analyses for FBG, FINS, TC, TG, LDL-C and HDL-C using Comprehensive Meta Analysis V3 (CMA) version 2.0. The mean differences were converted to standard mean differences (SMDs) and 95% confidence intervals (CIs), in accordance with the Cochrane Manual guidelines, to estimate effect sizes. This approach enabled the presentation of effect sizes from comparisons between experimental and control groups in forest plots. Heterogeneity was estimated using the Q statistic and the I^2^ test (Deeks et al., 2019). The Q statistic and I^2^ test were used to estimate heterogeneity. The Q statistic was used to identify heterogeneity. Each quartile is considered a heterogeneity interval, with values of 0–30%, >31–50%, >51–75%, and >75% indicating no heterogeneity, mild heterogeneity, moderate heterogeneity, and high heterogeneity, respectively, which were analyzed using a fixed-effects model for no heterogeneity and mild heterogeneity and a random-effects model for moderate and high heterogeneity. Additionally, publication bias was assessed through funnel plots (Peters et al., 2008) combined with Egger’s test (Egger et al., 1997) with *p* > 0.05 indicating no publication bias. The statistical significance level was set at α = 0.05.

Subgroup analyses were conducted to assess the effects of the following intervention-related factors: (1) exercise type (CET, RT, CT, INT, HYB); (2) frequency (3 *vs*. 5 sessions/week); (3) Supervision (supervised *vs*. unsupervised); (4) intervention duration (meta-regression). The exclusion of outliers was executed through the implementation of a sensitivity analysis (Fodor et al., 2018). Outliers were considered to be studies in which the 95% confidence interval (CI) of the individual effect size exceeded the 95% CI of the combined effect size (upper and lower bounds). Studies with effect sizes greater than 3 were considered outliers and were similarly excluded from the meta-analysis (Kadlec, Sainani & Nimphius, 2023).

## Results

### Literature selection

**Figure 1:**
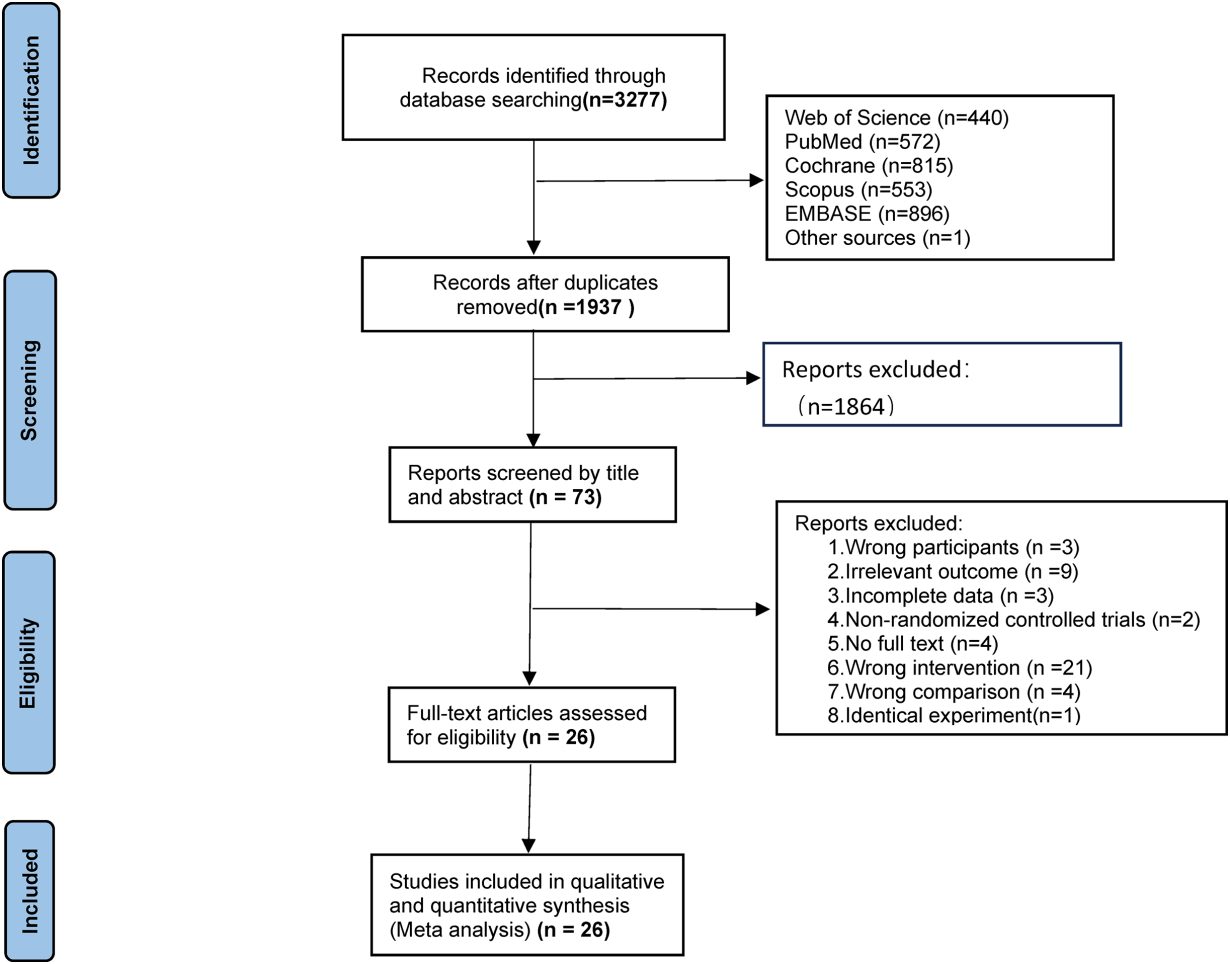
Flow diagram.

The initial search yielded 3,277 records (Web of Science: 440; PubMed: 572; Scopus: 553; Cochrane: 815; Embase: 396; reference lists: (1). After removing 1,350 duplicates, 1,927 records underwent title/abstract screening, retaining 73 for full-text review. Exclusions included: non-English articles (*n* = 2), non-exercise interventions (*n* = 14), ineligible populations (*n* = 3), non-compliant interventions (*n* = 7), inadequate control groups (*n* = 4), unavailable full texts (*n* = 4), missing outcomes (*n* = 9), non-RCT designs (*n* = 2), and duplicate datasets (*n* = 2). Ultimately, 26 studies were included (Fig. 1).

### Study characteristics

A synopsis of the study characteristics can be found in Table S3, and a list of the studies can be found in the Appendix S1. Studies spanned publication years 2006–2024, enrolling 984 participants (experimental groups: 576; control groups: 408). Females comprised 51.8% of participants, with two studies omitting gender data (Monteiro et al., 2015; Silva, Petroski & Pelegrini, 2014). Mean age ranged from 8.9 to 17 years, and mean BMI varied between 25.2 and 36.57 kg/m^2^. All 26 studies were randomized controlled studies. Exercise intervention modalities included CET (nine studies), CT (six studies), INT (six studies), HYB (10 studies), and RT (one study). Five of the studies had two exercise intervention groups (Monteiro et al., 2015; Meng et al., 2022; Khaliltahmasebi, Minasian & Hovsepian, 2022; González-Ruíz et al., 2022; Racil et al., 2016) including evaluations of CET *vs*. CT, CET *vs*. INT, and INT *vs*. HYB. Thus, a total of 31 exercise intervention groups and 26 conventional control groups were evaluated. All interventions were supervised: 16 studies used heart rate monitors for intensity control, one employed pedometers (Staiano et al., 2017) and others relied on certified trainers (Kim et al., 2007; Duft et al., 2020; Khaliltahmasebi, Minasian & Hovsepian, 2022; González-Ruíz et al., 2022; Staiano et al., 2018; Kim et al., 2011; Silva, Petroski & Pelegrini, 2014; Horner et al., 2015) with no unsupervised or remotely supervised trials identified. Intervention durations ranged from 4 to 26 weeks, with 81% (*n* = 21) lasting ≥12 weeks. Frequency was 3 sessions/week in 65% (*n* = 17) of studies.

### Risk-of-bias assessment and quality assessment

The results of the risk of bias assessment are presented in the Fig. S1. As the outcome indicators were derived from objective measurements, all studies were evaluated as low risk of bias in terms of blinded outcome assessment. Fourteen studies described the process of generating the random array in detail, while only 11 studies described the allocation concealment process. In addition, five studies had a dropout rate of more than 20%, and although the cause of dropout and the number of cases were described, they did not do an ITT analysis, which was evaluated as high risk of bias. One study did not describe the cause of dropout in detail, which was evaluated as unknown risk. In terms of selective reporting, four studies did not provide the registration platform of the study protocol and the registration number, which was rated as unknown risk of bias; and two studies did not report the negative and positive results in full, which was evaluated as high risk of bias; and two studies were at high risk of other biases, because the intervention and control groups’ baseline data were significantly different.

GRADE evidence quality was low for FBG and FINS, moderate for TC, TG, LDL-C, and HDL-C (Table S4), downgraded due to heterogeneity (I^2^ > 50%) and limited low-bias RCTs (<75%).

### Effects of intervention

**Figure 2:**
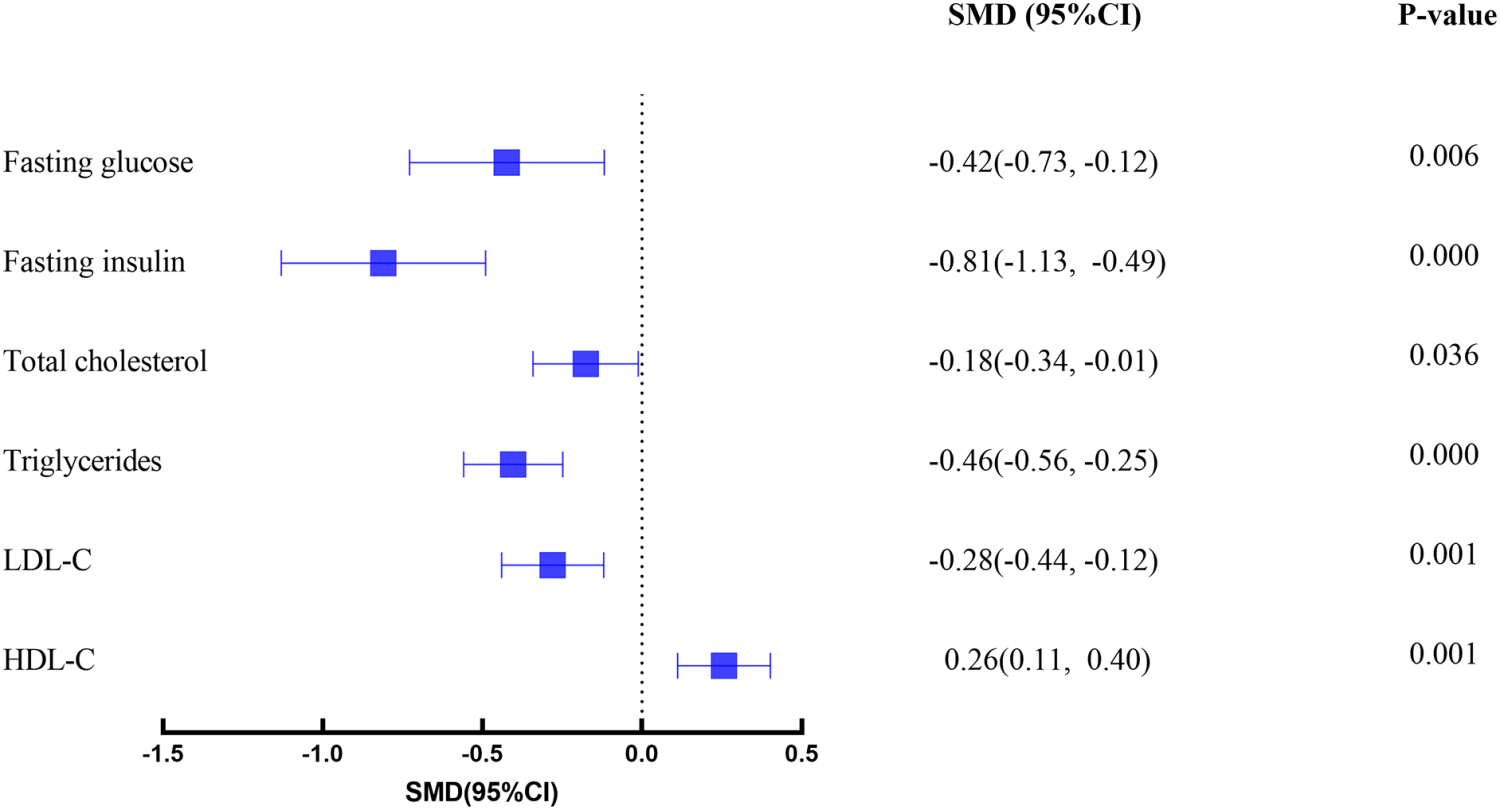
Forest plot.

As illustrated in Fig. 2, an overview of the primary effects for all glycemic and lipid outcomes is presented. The findings indicate the presence of small to moderate effects for exercise interventions when compared with usual care. The results of subgroup and meta-regression analyses for various exercise modalities, exercise frequency, supervision, and exercise length are delineated in Table 1.

**Table 1:** The results of meta-analyses.

Outcome	Heterogeneity				Categories	k	SMD [95% CI]	*p*
	*Q*	df	*p*	I^2^				
FBG (overall)	48.68	17	0.000	65.07		18	−0.42 [−0.73 to −0.12]	**0.006**
Mode	7.933	3	**0.047**	62.34	CET	5	−0.61 [−1.16 to −0.07]	**0.028**
				24.43	CT	2	0.30 [−0.23 to 0.82]	0.266
				77.19	HYB	6	−0.42 [−1.07 to 0.23]	0.206
				0.00	INT	5	−0.56 [−0.83 to −0.18]	**0.004**
Frequency	1.024	1	0.311	52.50	3 times/week	15	−0.51 [−0.67 to −0.18]	**0.003**
				76.78	5 times/week	3	−0.04 [−0.89 to 0.81]	0.929
Supervised or not	0.481	1	0.488	64.36	Supervised	13	−0.50 [−0.87 to −0.13]	**0.008**
				71.54	Nonsupervised	5	−0.25 [−0.84 to 0.33]	0.398
FINS (overall)	63.01	18	0.000	71.43		19	−0.81 [−1.13 to −0.49]	**0.000**
Mode	3.356	3	0.340	85.61	CET	6	−1.03 [−1.76 to −0.30]	**0.006**
				45.83	CT	3	−0.30 [−0.87 to 0.27]	0.299
				0.00	HYB	5	−0.70 [−1.05 to −0.35]	**0.000**
				65.02	INT	5	−0.98 [−1.66 to −0.30]	**0.005**
Frequency	0.977	1	0.323	47.48	3 times/week	16	−0.67 [−0.95 to −0.41]	**0.000**
				88.01	5 times/week	3	−1.29 [−2.45 to −0.14]	**0.028**
Supervised or not	0.276	1	0.600	77.52	Supervised	15	−0.84 [−1.26 to −0.43]	**0.000**
				0.00	Nonsupervised	4	−0.70 [−1.05 to −0.34]	**0.000**
TC (overall)	27.60	21	0.152	23.92		22	−0.18 [−0.34 to −0.01]	**0.036**
Mode	0.955	4	0.917	0.00	CET	7	−0.16 [−0.45 to 0.14]	0.291
				68.23	CT	4	−0.30 [−0.80 to 0.20]	0.426
				54.99	HYB	5	−0.22 [−0.73 to 0.43]	0.407
				35.24	INT	5	−0.13 [−0.62 to 0.37]	0.641
				0.00	RT	1	−0.51 [−1.20 to 0.18]	0.149
Frequency	0.883	1	0.347	24.02	3 times/week	19	−0.21 [−0.39 to −0.03]	**0.021**
				33.37	5 times/week	3	0. 00 [−0.40 to 0.41]	0.982
Supervised or not	2.336	1	0.126	29.82	Supervised	12	−0.30 [−0.52 to −0.07]	**0.009**
				5.16	Nonsupervised	10	−0. 04 [−0.28 to 0.20]	0.762
TG (overall)	32.44	22	0.07	32.18		22	−0.40 [−0.56 to −0.25]	**0.000**
Mode	3.65	4	0.456	49..57	CET	8	−0.42 [−0.80 to −0.14]	**0.029**
				53.08	CT	2	−0.37 [−0.86 to 0.12]	0.137
				0.00	HYB	7	−0.50 [−0.77 to −0.23]	**0.000**
				50.53	INT	5	−0.40 [−0.97 to 0.18]	0.173
				0.00	RT	1	0.22 [−0.47 to 0.90]	0.536
Frequency	0.04	1	0.84	38.12	3 times/week	20	−0.41 [−0.63 to −0.20]	**0.000**
				0.00	5 times/week	3	−0.39 [−0.80 to 0.02]	0.061
supervised or not	4.174	1	**0.04**	35.57	Supervised	15	−0.52 [−0.77 to −0.28]	**0.000**
				0.00	Nonsupervised	8	−0. 17 [−0.45 to 0.10]	0.216
LDL-C (overall)	36.93	21	0.02	43.14		22	−0.28 [−0.44 to −0.12]	**0.001**
Mode	8.45	4	**0.049**	45.97	CET	8	0.02 [−0.34 to 0.38]	0.897
				72.80	CT	2	−0.49 [−1.52 to 0.53]	0.098
				0.00	HYB	6	−0.60 [−0.91 to −0.30]	**0.000**
				37.12	INT	5	−0.55 [−1.06 to −0.04]	**0.035**
				0.00	RT	1	0.00 [−0.67 to 0.68]	0.994
Frequency	0.17	1	0.68	43.69	3 times/week	19	−0.29 [−0.53 to −0.05]	**0.005**
				51.69	5 times/week	3	−0.42 [−1.02 to 0.17]	0.164
supervised or not	0.825	1	0.36	56.11	Supervised	14	−0.38 [−0.68 to −0.07]	**0.017**
				0.00	Nonsupervised	8	−0. 18 [−0.46 to 0.09]	0.191
HDL-C (overall)	33.03	22	0.06	33.39		22	0.26 [0.11–0.40]	**0.001**
Mode	0.57	4	0.966	1.25	CET	8	0.26 [−0.02 to 0.53]	0.064
				65.45	CT	2	0.39 [−0.93 to 1.72]	0.566
				47.66	HYB	6	0.23 [−0.12 to 0.64]	0.185
				9.42	INT	5	0.49 [−0.03 to 0.77]	0.070
				0.00	RT	1	0.07 [−0.49 to 0.62]	0.817
Frequency	0.08	1	0.78	27.15	3 times/week	20	0.26 [0.06–0.47]	**0.013**
				0.00	5 times/week	3	0.31 [−0.11 to 0.74]	0.144
Supervised or not	0.17	1	0.68	26.05	Supervised	13	0.27 [0.02–0.52]	**0.039**
				21.92	Nonsupervised	10	0.28 [−0.01 to 0.57]	0.058
**Covariat (Length)**			***p***		**Coefficient**	**Standard error**	**Lower limit**	**Upper limit**
FBG			0.935		−0.0021	0.026	−0.053	0.049
FINS			0.783		0.0069	0.025	−0.424	0.056
TC			0.164		0.0229	0.017	−0.009	0.055
TG			0.728		−0.0156	0.016	−0.036	0.025
LDL-C			0.816		−0.0043	0.018	−0.040	0.032
HDL-C			**0.042**		0.0358	0.018	0.001	0.070

**Note:**

CET, continuous endurance training; CON, control; CT, combined training; FBG, fasting blood glucose; FINS, fasting insulin; HDL, high-density lipoprotein; HYB, hybrid-type training; INT, interval training; LDL, low-density lipoprotein; RT, resistance training; TC, total cholesterol; TG, triglycerides. Bold entries indicate *p* < 0.05.

### Fasting blood glucose

A total of 15 studies (18 intervention groups and 15 control groups) explored the effect of exercise as an intervention on FBG in obese adolescents. The analysis revealed that compared with the control group, the FBG in the exercise intervention group decreased significantly (SMD: −0.42 95% CI [−0.73 to −0.12], *p* = 0.006). However, moderate heterogeneity was observed (I^2^ = 65.07%, *p* < 0.001).

Subgroup analysis revealed that the impact of distinct exercise modalities on FBG in obese adolescents exhibited significant variation (*Q* = 7.933, df = 3, *p* = 0.047). Among these modalities, CET demonstrated the most substantial effect on FBG in obese adolescents (SMD: −0.61 [−1.16 to −0.07]), followed by INT (SMD: −0.56 [−0.83 to 0.18]). Conversely, CT (*p* = 0.266) and HYB (*p* = 0.206) did not demonstrate a significant impact on FBG levels in obese adolescents. Two additional subgroup analyses revealed that training three times per week (*p* = 0.003) under supervised (*p* = 0.008) conditions was superior to training five times per week (*p* = 0.398) under unsupervised (*p* = 0.929) conditions. Further meta-regression showed that intervention length did not affect fasting glucose in overweight adolescents (*p* = 0.935).

### Fasting insulin

A total of 17 studies (19 intervention groups and 17 control groups) explored the effect of exercise as an intervention on FINS in obese adolescents. Sensitivity analysis identified two outliers: SMD = 7.264 (Wong et al., 2018) and SMD = −7.004 (Bharath et al., 2018), After removing these outliers, the overall effect remained the same, and a statistically significant decrease in FINS was observed in the exercise intervention group compared to the control group (SMD: −0.81 [−1.13 to −0.49], *p* < 0.001). However, moderate heterogeneity was observed (I^2^ = 71.94%, *p* < 0.001). Subgroup analysis and meta-regression results demonstrated that all had a significant effect on FINS in obese adolescents, with the exception of CT (*p* = 0.299). Furthermore, intervention frequency (*p* = 0.32), supervision (*p* = 0.60), and intervention length (*p* = 0.78) were found to have no significant effect on the results.

### Total cholesterol

A total of 17 studies (20 intervention and 17 control groups) explored the effect of exercise as an intervention on TC in obese adolescents. Sensitivity analysis identified one outlier: SMD = 3.943 (Staiano et al., 2018). After removing the outlier the overall effect remained unchanged, and compared to the control group, the TC in the exercise intervention group was statistically significantly reduced (SMD: −0.18 [−0.34 to −0.01], *p* = 0.045); heterogeneity was low and not significant (I^2^ = 23.92%, *p* = 0.15). Subgroup analysis and meta-regression results showed that exercise mode (*p* = 0.95) and intervention length (*p* = 0.16) did not significantly affect the results, and supervised (*p* = 0.009) training once a week was also better than unsupervised (*p* = 0.762) training.

### Triglycerides

A total of 18 studies (23 intervention and 18 control groups) explored the effect of exercise as an intervention on TG in obese adolescents. Compared to the control group, TG were statistically significantly reduced in the exercise intervention group (SMD: −0.46 [−0.56 to −0.25], *p* < 0.001). The study revealed moderate heterogeneity (I^2^ = 32.18%, *p* = 0.07). Subgroup analysis and meta-regression results indicated that, in terms of exercise mode, Only CET (*p* = 0.029) and HYB (*p* = 0.00) significantly reduced triglyceride levels in obese adolescents. Furthermore, training under supervised conditions (*p* = 0.000) was found to be more effective than training under unsupervised conditions (*p* = 0.216).

### Low-density lipoprotein cholesterol

A total of 18 studies (23 intervention and 18 control groups) explored the effect of exercise as an intervention on LDL-C in overweight adolescents. The analysis revealed a statistically significant reduction in LDL-C levels in the exercise intervention group compared to the control group (SMD: −0.28 [−0.44 to −0.12], *p* = 0.01). However, moderate heterogeneity was observed (I^2^ = 43.14%, *p* = 0.02).

Subgroup analysis and meta-regression results showed that different exercise modes affect LDL-C in overweight adolescents (*p* = 0.049), among which HYB (*p* = 0.00) and INT (*p* = 0.04) significantly reduced Conversely, CET (*p* = 0.897), CT (*p* = 0.098), and RT (*p* = 0.994) demonstrated no significant impact on LDL-C levels in obese adolescents. In a similar vein, the study found that supervised exercise (*p* = 0.02) was superior to unsupervised training (*p* = 0.191). The intervention length (*p* = 0.816) did not significantly affect the results.

### High-density lipoprotein cholesterol

A total of 18 studies (23 intervention and 18 control groups) investigated the effect of exercise as an intervention on HDL-C in overweight adolescents. The analysis revealed that HDL-C was statistically significantly higher in the exercise intervention group than in the control group (SMD: 0.26 [0.11–0.40], *p* < 0.001). The analysis also reported low and non-significant heterogeneity (I^2^ = 33.39%, *p* = 0.06).

Subgroup analysis and meta-regression results demonstrated that different exercise modes had no impact on HDL-C in overweight adolescents (*p* = 0.966). Additionally, the analysis revealed that supervised exercise (*p* = 0.039) was more effective than unsupervised exercise (*p* = 0.058). Furthermore, the intervention length (*p* = 0.042) was found to have a significant impact on HDL-C levels in overweight adolescents. Specifically, the results indicated that longer intervention periods were associated with more substantial increases in HDL-C levels.

### Sensitivity and publication bias

We performed a series of sensitivity analyses to ascertain the association between potential sources of heterogeneity. The effect sizes of three outliers were excluded in two outcome measures (Staiano et al., 2018; Bharath et al., 2018; Wong et al., 2018). The results of the processing indicate that the data is insensitive, which did not significantly interfere with the results of this meta-analysis, indicating that this meta-analysis has good stability and reliability. The funnel plot test (Fig. S3) revealed a roughly symmetrical distribution, indicating an absence of statistically significant publication bias. Furthermore, the Egger’s test (Table S5) yielded similar conclusions, confirming the absence of publication bias in the study (*p* > 0.05).

## Discussion

This systematic review and meta-analysis of 26 RCTs involving 984 overweight and obese adolescents provides robust evidence that exercise interventions significantly improve both glycemic (FBG, FINS) and lipid metabolism (TC, TG, LDL-C, HDL-C). CET demonstrated greater efficacy in improving blood glucose parameters, while HYB demonstrated superior efficacy in optimizing lipid profiles. Critically, supervised sessions administered three times weekly were identified as the optimal regimen for achieving metabolic benefits, underscoring the importance of structured implementation in this population.

The observed reduction in FBG (SMD: −0.42 [−0.73 to −0.12]) aligns with prior meta-analyses, though the effect sizes in our study were slightly smaller than those reported by Li et al. (2024) (range: −1.24 to −0.32). This discrepancy may stem from our stringent inclusion criteria, which excluded studies combining exercise with dietary or psychological interventions, thereby isolating the independent effects of physical activity. With regard to the FINS indicator, a meta-analysis demonstrated the beneficial effect of exercise interventions on FINS levels in obese adolescents, and found that CET effectively reduced fasting insulin, while CT did not affect FINS levels (Liang et al., 2021). This is also the same as our results. However, it should be noted that certain studies have produced results that are not consistent with our own. A meta-analysis demonstrated that, compared with the control group, physical exercise only reduced FINS and insulin resistance, but did not affect the level of FBG (Marson et al., 2016). A similar finding reported that INT reduced FINS and insulin resistance in obese children but had no effect on FBG (Zhu et al., 2021). Another study also reported that exercise intervention did not significantly improve FBG in obese adolescents (Lee, 2021). These differences may be due to two factors: on the one hand, we have included more updated research results than previous articles, and on the other hand, they may be due to different inclusion criteria. In terms of intervention, we have included five types of exercise, while other studies have included one to three. In addition, our inclusion criteria require exercise to be the only intervention, which is also different from some studies.

Regarding the results of exercise on lipid metabolism in obese adolescents, our findings are similar to those of the meta-analysis by Li & Chen (2021). The exercise intervention demonstrated a significant improvement in TC, TG, and LDL-C levels in obese adolescents. However, Li & Chen (2021) did not find a significant increase in HDL-C, which differs from the results of our study. It is important to note that several meta-analyses have reached conclusions that are contradictory to those of this study. For instance, Pozuelo-Carrascosa et al. (2018a) demonstrated that school-based physical education programs may not be adequate to substantially enhance lipid metabolism in obese adolescents, exhibiting no significant impact on TC, TG, LDL-C, and HDL-C (all *p* > 0.05). This discrepancy may be attributable to methodological limitations in prior studies, such as insufficient details on intervention implementation and a lack of adherence reporting in most trials.

The efficacy of exercise in modulating glycolipid metabolism in overweight and obese adolescents depends on multiple factors, including exercise modality, supervision, frequency, and duration. Our findings suggest that CET is most effective for improving blood glucose parameters, whereas HYB yields greater benefits for lipid metabolism. These observations are partially supported by Jamka et al. (2022) who found that comprehensive exercise regimens outperformed CET or RT alone in improving TC, LDL-C, HDL-C, and TG levels, while CET more effectively reduced FBG and FINS. Similarly, Batrakoulis et al. (2022) compared the effects of different exercise modalities (CET, INT, RT, CT, and HYB) on cardiometabolic parameters in overweight and obese subjects. The findings of the meta-analysis indicated that HYB exhibited the most efficacy in reducing FINS concentrations and enhancing HDL-C levels. Concurrently, CT demonstrated the most significant impact in reducing FINS and LDL-C levels, while INT revealed the greatest potential in modulating TG levels (Batrakoulis et al., 2022). However, it should be emphasized that the meta-analysis included studies conducted in overweight and obese adult subjects, which may explain the differences in the results obtained in this study. In a related meta-analysis, Liang et al. (2021) examined the effects of CET, RT, and CT on metabolic syndrome parameters and cardiovascular risk factors through a network meta-analysis to determine the most effective method for improving metabolic syndrome and preventing cardiovascular disease. The Surface Under the Cumulative Ranking curve (SUCRA) reveals that CT exhibited the greatest efficacy in enhancing FINS and TC and TG levels, RT demonstrated the strongest impact on LDL-C levels, and CET showed the most significant improvement in FBG levels. These findings are largely consistent with the research results of the present study.

The observed discrepancy in the effectiveness of different exercise forms may be attributed to their impact on weight loss and alterations in body composition. The reduction of visceral fat has been shown to impact cardiometabolic parameters, and abdominal obesity is strongly associated with impaired blood lipid levels due to increased visceral fat accumulation (Bays et al., 2008). In comparison with other forms of exercise, HYB has been demonstrated to be the most efficacious in terms of weight reduction and possesses the greatest potential to decrease adipose tissue in obese subjects without comorbidities (Batrakoulis et al., 2022). This also explains the significant role of HYB in improving lipid metabolism in obese adolescents. The studies included in our meta-analysis indicate that HYB tend to be longer, which may be another reason for their significant improvement in blood lipid metabolism. Exercise can increase the consumption of stored body fat and glycogen, reduce energy intake and hepatic glycogenolysis, and improve the sensitivity of the liver, adipose tissue and skeletal muscle tissue to insulin, thereby improving body composition. According to Davidson et al. (2009), CET may lead to greater energy expenditure and, consequently, a more substantial reduction in FBG levels in overweight and obese adolescents.

Our study demonstrates that supervision enhances the impact of exercise intervention on glycolipid metabolism in overweight and obese adolescents. This finding aligns with the results of most studies, and the definition of supervision in this study is to use a heart rate monitor or step count monitor to ensure that participants achieve the required load intensity and load, so it has higher credibility. Our study also found that exercise 5 times per week did not yield greater benefits than exercise 3 times per week. Reducing caloric intake was the key factor influencing weight loss and the improvement of cardiometabolic parameters, however, in our meta-analysis, only studies in which participants were instructed not to alter their dietary habits during the intervention period were included. Although higher exercise frequency increases energy expenditure, the unhealthy eating habits of obese adolescents may lead to an increase in energy intake, which offsets these gains. The duration of the intervention may also have affected the results of the meta-analysis. To address this, a meta-regression analysis was conducted, which revealed that, with the exception of HDL-C, the duration of the intervention had no discernible impact on the outcomes of the meta-analysis. These findings suggest a challenge in sustaining elevated levels of compliance in prolonged intervention studies, which may be associated with diminished motivation and elevated rates of attrition. Additionally, a “ceiling effect” may be present, indicating that exercise interventions may only be able to normalize blood glucose and lipid indicators in overweight and obese adolescents.

The present review is limited in scope due to its restriction to English-language publications. Moreover, the majority of the included studies did not report on participant compliance, and there was an absence of follow-up, precluding the determination of long-term effects of exercise interventions.

Future research should extend the scope to include additional publications in this field and explore in greater depth the effects of exercise on the glycolipid metabolism of overweight and obese adolescents. Such as the long-term effects of exercise interventions, the influence of gender differences on the effects of exercise interventions, and the formulation of more detailed exercise prescriptions for improving the glycolipid metabolism of overweight and obese adolescents.

## Conclusion

Our meta-analysis suggests that exercise interventions can improve glycolipid metabolism in overweight and obese adolescents, as indicated by reductions in FBG, FINS, TC, TG, LDL-C and increase in HDL-C. Compared with other forms of exercise, CET demonstrated more significant improvements in blood glucose indicators, while HYB had greater advantage in lipid metabolism. Overall, the optimal approach to enhance glycolipid metabolism in obese adolescents appears to be supervised exercise 3 times per week. The findings of this study provide evidence for clinicians and other healthcare professionals (*e.g*., exercise physiologists, physical therapists) to guide obese adolescents in engaging in exercise, in order to prevent worsening obesity and glucose-lipid metabolic imbalances. However, larger-scale studies are needed to determine the effectiveness of different exercise programs (including types of exercise and intervention duration) in improving glycolipid metabolism in overweight and obese adolescents.

## Supplemental Information

10.7717/peerj.19365/supp-1 Supplemental Information 1 PRISMA checklist.

10.7717/peerj.19365/supp-2 Supplemental Information 2 Search strategy, characteristics of each type of exercise, characteristics of the included studies, risk of bias assessment, quality assessment, forest plots, funnel.

## Additional Information and Declarations

### Data Availability

The following information was supplied regarding data availability:

This is a systematic review/meta-analysis.
