# Supplementary material for: Effects of exercise on glycolipid metabolism in adolescents with overweight and obesity: a systematic review and meta-analysis of 26 randomized controlled trials
Source: PeerJ. 2025 Apr 23;13:e19365. doi: 10.7717/peerj.19365 (PMC12032957; doi:10.7717/peerj.19365)
Supplement: Supplemental Information 2 [file peerj-13-19365-s002.docx]

**Supplementary Material**

**Table S1.** Search strategy of PubMed, Embase, Cochrane, Web of Science and EBSCO.

| **Database** | **Search Terms** |
| --- | --- |
| **PubMed [Title/Abstract]** | (child[MeSH Terms] OR kids OR Adolescents OR Adolescence OR Youths OR Teen* ) AND (obesity[MeSH Terms] OR obese OR overweight) AND (exercise [MeSH Terms] OR training OR sport* OR “aerobic training” OR “endurance training” OR “moderate-intensity continuous training” OR “resistance training” OR “weight training” OR “strength training” OR “combined training” OR “high-intensity intermittent exercise” OR “moderate-intensity intermittent exercise” OR “interval exercise” OR “interval training” OR “sprint interval training” OR MIIT OR HIIT OR “multimodal training” OR “multi-modal training” OR “concurrent training” OR crossfit OR “high-intensity functional training” OR HIFT OR “cardio-resistance training” OR “integrated neuromuscular training” OR “small-sided games”) AND (“Glycolipid Metabolism” OR "glucose and lipid Metabolism" OR metabol* OR Cardiometabolic OR “lipid*” OR cholesterol OR “TC” OR triglycerides OR “low-density lipoprotein” OR “high-density lipoprotein” OR insulin OR “blood glucose”) AND (“randomized controlled trail” OR “controlled trail” OR randomized OR trail) NOT (animals OR elderly OR aged OR older adults) |
| **Embase [Title/Abstract]** | (child [Emtree term] OR kids OR Adolescents OR Adolescence OR Youths OR Teen* ) AND (obesity [Emtree term] OR obese OR overweight) AND (training [Emtree term] OR exercise OR sport* OR “aerobic training” OR “endurance training” OR “moderate-intensity continuous training” OR “resistance training” OR “weight training” OR “strength training” OR “combined training” OR “high-intensity intermittent exercise” OR “moderate-intensity intermittent exercise” OR “interval exercise” OR “interval training” OR “sprint interval training” OR MIIT OR HIIT OR “multimodal training” OR “multi-modal training” OR “concurrent training” OR crossfit OR “high-intensity functional training” OR HIFT OR “cardio-resistance training” OR “integrated neuromuscular training” OR “small-sided games”) AND (“Glycolipid Metabolism” OR "glucose and lipid Metabolism" OR metabol* OR Cardiometabolic OR “lipid*” OR cholesterol OR “TC” OR triglycerides OR “low-density lipoprotein” OR “high-density lipoprotein” OR insulin OR “blood glucose”) AND (“randomized controlled trail” OR “controlled trail” OR randomized OR trail) |
| **Cochrane [Title/Abstract/ keywords]** | (child[MeSH Terms] OR kids OR Adolescents OR Adolescence OR Youths OR Teen* ) AND (obesity[MeSH Terms] OR obese OR overweight) AND (exercise [MeSH Terms] OR training OR sport* OR “aerobic training” OR “endurance training” OR “moderate-intensity continuous training” OR “resistance training” OR “weight training” OR “strength training” OR “combined training” OR “high-intensity intermittent exercise” OR “moderate-intensity intermittent exercise” OR “interval exercise” OR “interval training” OR “sprint interval training” OR MIIT OR HIIT OR “multimodal training” OR “multi-modal training” OR “concurrent training” OR crossfit OR “high-intensity functional training” OR HIFT OR “cardio-resistance training” OR “integrated neuromuscular training” OR “small-sided games”) AND (“Glycolipid Metabolism” OR "glucose and lipid Metabolism" OR metabol* OR Cardiometabolic OR “lipid*” OR cholesterol OR “TC” OR triglycerides OR “low-density lipoprotein” OR “high-density lipoprotein” OR insulin OR “blood glucose”) AND (“randomized controlled trail” OR “controlled trail” OR randomized OR trail) |
| **Web of Science** | TS=(child* OR kids OR Adolescents OR Adolescence OR Youths OR Teen* ) AND TS=(obesity OR obese OR overweight) AND TS=( exercise* OR “physical activity” OR training OR sport* OR “aerobic training” OR “endurance training” OR “moderate-intensity continuous training” OR “resistance training” OR “weight training” OR “strength training” OR “combined training” OR “high-intensity intermittent exercise” OR “moderate-intensity intermittent exercise” OR “interval exercise” OR “interval training” OR “sprint interval training” OR MIIT OR HIIT OR “multimodal training” OR “multi-modal training” OR “concurrent training” OR crossfit OR “high-intensity functional training” OR HIFT OR “cardio-resistance training” OR “integrated neuromuscular training” OR “small-sided games”) AND TS=(“Glycolipid Metabolism” OR "glucose and lipid Metabolism" OR metabol* OR Cardiometabolic OR “lipid*” OR cholesterol OR “TC” OR triglycerides OR “low-density lipoprotein” OR “high-density lipoprotein” OR insulin OR “blood glucose” OR BMI OR “body mass index” OR “body mass” OR “body composition”) AND TS=(“randomized controlled trail” OR “controlled trail” OR randomized OR trail) NOT TS=(animals OR elderly OR aged OR older adults) |
| **EBSCO**  **[Abstract]** | (child OR kids OR Adolescents OR Adolescence OR Youths OR Teen* ) AND (obesity OR obese OR overweight) AND (exercise OR training OR sport* OR “aerobic training” OR “endurance training” OR “moderate-intensity continuous training” OR “resistance training” OR “weight training” OR “strength training” OR “combined training” OR “high-intensity intermittent exercise” OR “moderate-intensity intermittent exercise” OR “interval exercise” OR “interval training” OR “sprint interval training” OR MIIT OR HIIT OR “multimodal training” OR “multi-modal training” OR “concurrent training” OR crossfit OR “high-intensity functional training” OR HIFT OR “cardio-resistance training” OR “integrated neuromuscular training” OR “small-sided games”) AND (“Glycolipid Metabolism” OR "glucose and lipid Metabolism" OR metabol* OR Cardiometabolic OR “lipid*” OR cholesterol OR “TC” OR triglycerides OR “low-density lipoprotein” OR “high-density lipoprotein” OR insulin OR “blood glucose”) AND (“randomized controlled trail” OR “controlled trail” OR randomized OR trail) |

| **Type of exercise** | **Definition** |
| --- | --- |
| **CET** | Time: 30–60 min per session |
|  | Intensity: >45% VO2 max or >50% HRR or >65% HRmax |
|  | Type: Any continuous traditional mode of aerobic training only (such as walking, running, cycling, rowing, swimming, aerobics, elliptical exercise, and stepping exercise) |
| **INT** | Gross exercise time: 20–30 min per session |
|  | Intensity: >65% VO2max or >65% HRR or >75% HRmax |
|  | Type: Any intermittent traditional mode of interval training only, including single-component MIIT and HIIT (such as walking, running, cycling, rowing, swimming, elliptical exercise, and stepping exercise) |
| **RT** | Gross exercise time: 30–60 min per session |
|  | Intensity: ≥50% 1RM |
|  | Type: Any mode of resistance training, including circuit-based programs (such as free weights, weights machines, and resistance bands) |
| **CT** | A combination of CET and RT |
| **HYB** | Gross exercise time: 30–45 min per session |
|  | Intensity: >65% VO2 max or >65% HRR or >75% HRmax |
|  | Type: Any comprehensive multi-component exercise mode engaging both the cardiovascular and the musculoskeletal system throughout a single exercise session using both muscle-strengthening and dynamic cardiovascular exercises (such as small-sided games in recreational sports, high-intensity functional training, integrated neuromuscular training, exergaming and multimodal training) |
| **CON** | No exercise |

**Table S2.** Characteristics of each type of exercise.

**Abbreviations:** CET, indicates continuous endurance training; CON, control; CT, combined training; HIIT, high-intensity interval training; HRmax, maximum heart rate; HRR, heart rate reserve; HYB, hybrid-type training; INT, interval training; MIIT, moderate-intensity interval training; RM, repetition maximum; RT, resistance training; VO2max, maximal oxygen uptake.

**Table S3.** Characteristics of the included studies.

| **study** | **Sample size**  **(N/F)** | **Age (mean±[SD])** | **BMI (kg/m2)** | **Duration (wks) & frequency (days/wk)** | **Exercise category** | **exercise details** | **supervised** | **Outcomes** | **adherence** | |
| --- | --- | --- | --- | --- | --- | --- | --- | --- | --- | --- |
| Meyer 2006[1] | 67/16 | 13.7 ± 2.1 | 29.10 ± 4.83 | 26/3 | CON | No exercise | supervised | FINS, LDL, HDL, TG | ≥92.3% |  |
|  |  |  |  |  | CET | 60 min swimming and aqua aerobic training or walking with no intensity prescribed |  |  |  |  |
| Kim 2007[2] | 26/0 | 17 ± 0.11 | 29.50 ± 0.4 | 6/5 | CON | No exercise | nonsupervised | FINS, TC, FBG, TG, LDL, HDL | NA |  |
|  |  |  |  |  | INT | 1 & 1.5 & 2 & 2.5 & 3 & 4 min of exercise (60-90 jumps/min), 30 secs of rest for each set |  |  |  |  |
| Lee 2008[3] | 17/0 | 11 ± 0 | 29.1 ± 1.2 | 12/3 | CON | No exercise | supervised | FINS | NA |  |
|  |  |  |  |  | CT | 35min 55-75% MHR aerobic exercise & 50min 70% RM Rubber band exercises |  |  |  |  |
| Lambert 2009[4] | 44/16 | 8.9 ± 1.5 | 28.5 ± 1.8 | 13/3 | CON | No exercise | supervised | TC, LDL, HDL, TG, FBG, FINS | ≥83% |  |
|  |  |  |  |  | CT | 30min 55-65% VO2max + 30min 2sets 15 repetitions body weight RT |  |  |  |  |
| Seo 2012[5] | 20/0 | 14.7 ± 0.7 | 28.80 ± 1.70 | 8/3 | CON | No exercise | supervised | TC, LDL, HDL, TG, FBG, FINS | ≥87.5% |  |
|  |  |  |  |  | CET | 60 mins 40 - 60% HRR Yoga Training |  |  |  |  |
| Davis 2011[6] | 26/26 | 14 ± 1.1 | 34.25 ± 5.02 | 16/3 | CON | No exercise | supervised | FBG, FINS | ≥87.5% |  |
|  |  |  |  |  | HYB | 60-90mins 70-85% HHR 6-8 intermittent multicomponent exercise |  |  |  |  |
| Castro 2020[7] | 37/18 | 14.48 ± 1.05 | 28.95 ± 3.44 | 12/3 | CON | No exercise | nonsupervised | TC, FBG, FINS | ≥80% |  |
|  |  |  |  |  | CT | 30 mins RT leg press, leg extension machine, leg flexion machine etc.+ 30mins CET 50- 85% VO2peak walking or running. |  |  |  |  |
| Monteiro 2015[8] | 48/NA | 11 ± 3.2 | 31.39 ± 3.67 | 20/3 | CON | No exercise | supervised | TC, TG, LDL, HDL | NA |  |
|  |  |  |  |  | CET | 60mins 65-85 % VO2peak walking and running |  |  |  |  |
|  |  |  |  |  | CT | 30mins CET & 55-75% 1RM low rowing, bench press, squat rack, seated lat pull-down etc. |  |  |  |  |
| Tas 2023[9] | 37/21 | 15.2 ± 1.4 | 36.57 ± 5.95 | 4/3 | CON | No exercise | supervised | TC, LDL, HDL, TG, FBG, FINS | 97% |  |
|  |  |  |  |  | INT | 80-90% VO2max 10 1-min intervals using exercise with 2 min of recovery time (walking) between each effort |  |  |  |  |
| Meng 2022[10] | 45/0 | 11.2 ± 0.7 | 25.2 ± 1.0 | 12/3 | CON | No exercise | supervised | TC, LDL, HDL, TG, FBG, FINS | 100% |  |
|  |  |  |  |  | CET | 30mins 50-70%HHR running |  |  |  |  |
|  |  |  |  |  | INT | 2 sets of 8t 15-s bouts(80 ~ 90% HHR) separated by eight 15-s recovery bouts (40 ~ 60% HHR) 3-min rest between two sets |  |  |  |  |
| Khaliltahmasebi 2022[11] | 38/0 | 12 ± 1 | 26.6 ± 2.3 | 12/3 | CON | No exercise | nonsupervised | TC, TG, LDL, HDL | ≥91.6% |  |
|  |  |  |  |  | INT | 4 sets 85-100% HYB & active recovery 5 min rest with 15‑min warm‑up & 15‑min cool‑down exercises |  |  |  |  |
|  |  |  |  |  | HYB | 90 mins shuttle run & futsal & resistance exercises |  |  |  |  |
| Bautista 2022[12] | 120/81 | 13 ± 2.1 | 32.3 ± 5.3 | 26/3 | CON | No exercise | nonsupervised | TC, LDL, HDL, TG, FBG, FINS | ≥76.3% |  |
|  |  |  |  |  | CET | 60mins 55-75% HHR training curriculum |  |  |  |  |
|  |  |  |  |  | HYB | 60mins 75–85% HHR includes basic sports games, traditional games, and other outdoor activities |  |  |  |  |
| Son 2020[13] | 48/48 | 15 ± 1 | 26 ± 2 | 12/5 | CON | No exercise | supervised | FINS，FBG | NA |  |
|  |  |  |  |  | CET | 50mins 40-70% HHR Jump rope exercise |  |  |  |  |
| Bharath 2018[14] | 20/20 | 14.7 ± 1 | 30 ± 2 | 12/5 | CON | No exercise | supervised | FINS, FBG | NA |  |
|  |  |  |  |  | CT | 30mins 50-70% HRR running & 15-20 receptions resistance bands perform exercises for the upper and lower limbs |  |  |  |  |
| Wong 2018[15] | 30/30 | 15.2 ± 1.2 | 27 ± 2.1 | 12/3 | CON | No exercise | supervised | FINS, FBG | NA |  |
|  |  |  |  |  | CT | 30mins resistant band exercises & 30mins 50-70HRR treadmill walking |  |  |  |  |
| Staiano 2018[16] | 46/21 | 11.2 ± 0.8 | 27.8 ± 3.2 | 24/3 | CON | No exercise | nonsupervised | TC, LDL, HDL, TG, FBG, | ≥88.5% |  |
|  |  |  |  |  | HYB | 60mins moderate-to-vigorous physical activity by 4 exergames |  |  |  |  |
| Zehsaz 2016[17] | 32/0 | 10.6 ± 0.9 | 29.0 ± 1.8 | 16/3 | CON | No exercise | supervised | TC, LDL, HDL, TG, FBG, FINS | NA |  |
|  |  |  |  |  | HYB | 60mins exercise including 65%-85% HRmax aerobic exercises & resistance training & game activities such as soccer |  |  |  |  |
| Staiano 2022[18] | 41/41 | 16 ± 1.4 | 30.2 ± 4.7 | 12/3 | CON | No exercise | supervised | TC, LDL, HDL, TG, FBG, FINS | ≥75% |  |
|  |  |  |  |  | HYB | 60mins group-based exergaming |  |  |  |  |
| Vasconcellos 2016[19] | 20/6 | 14.3 ± 1.4 | 31.6 ± 5.0 | 12/3 | CON | No exercise | supervised | TC, LDL, HDL, TG, FBG, FINS | NA |  |
|  |  |  |  |  | HYB | 60mins 85% HRmax soccer games |  |  |  |  |
| Racil 2016[20] | 68/68 | 16.6 ± 1.3 | 33.2 ± 5.6 | 12/3 | CON | No exercise | supervised | FINS，FBG | NA |  |
|  |  |  |  |  | INT | 6-8 bouts of 30-s 100% VO2max runs at with 30 s recovery between bouts at 50% V̇O2peak, The two blocks were separated by a 4-min passive recovery period |  |  |  |  |
|  |  |  |  |  | HYB | 60-90 mins plyometric exercises (medicine ball single-leg dip, hurdle hops etc.) |  |  |  |  |
| Racil 2013[21] | 34/34 | 15.9 ± 0.3 | 30.8 ± 1.6 | 12/3 | CON | No exercise | supervised | TC, LDL, HDL, TG, FBG, FINS | NA |  |
|  |  |  |  |  | INT | 100%-110% MAS 6-8 repetitions 30 seconds running + 30 seconds active recovery + 4 minutes of recovery time |  |  |  |  |
| Horner 2015[22] | 81/44 | 14.7 ± 1.8 | 34.3 ± 4.4 | 13/3 | CON | No exercise | nonsupervised | TC, TG, LDL, HDL | NA |  |
|  |  |  |  |  | CET | 60 mins 60-75% VO2max Treadmills or stationary bicycle exercise |  |  |  |  |
|  |  |  |  |  | RT | 10 exercises for the whole body (2 sets 8-12 repetitions) |  |  |  |  |
| Silva 2014[23] | 14/NA | 14 ± 3.1 | 28.9 ± 2.7 | 12/3 | CON | No exercise | nonsupervised | TC, TG, LDL, HDL | ≥75% |  |
|  |  |  |  |  | CET | 40mins Lactate threshold intensity cycle ergometer training |  |  |  |  |
| McCormack 2014[24] | 18/13 | 13.0 ± 1.9 | 37.0 ± 7.1 | 8/3 | CON | No exercise | supervised | FINS, TC, FBG | ≥75% |  |
|  |  |  |  |  | HYB | 60mins 60-80% HRR GameBike & Stretching and resistance training from Exergaming |  |  |  |  |
| Kim 2011[25] | 30/0 | 17.0 ± 0.1 | 28.60 ± 3.41 | 12/5 | CON | No exercise | nonsupervised | TC, LDL, HDL, TG, FBG, FINS | NA |  |
|  |  |  |  |  | HYB | 50mins exercise Including running, stretching, jumping rope, badminton, basketball and dance. |  |  |  |  |
| Sun 2011[26] | 42/17 | 13.6 ± 0.7 | 27.1 ± 3.1 | 10/5 | CON | No exercise | supervised | TC, TG, LDL, HDL | NA |  |
|  |  |  |  |  | HYB | 60mins 40%-60% VO2max exercise including moderate-speed running, skipping rope, and group activities such as basketball, volleyball, and badminton |  |  |  |  |

**Abbreviations:** N, number; F, female; NA, not Available; CON, control; CET, continuous endurance training; INT, interval training; RT, resistance training; CT, combined training; HYB, hybrid-type training; RM, repetition maximum; VO2max, maximal oxygen uptake; VO2peak, peak oxygen uptake; reps, repetitions; HRmax, maximum heart rate; MAS, Maximum Aerobic Speed; HRR, heart rate reserve; FBG, fasting blood glucose; FINS, fasting insulin; TC, Total cholesterol; TG, Triglycerides; HDL, high-density lipoprotein; LDL, low-density lipoprotein.

**Figure S1.** Risk of bias assessment.


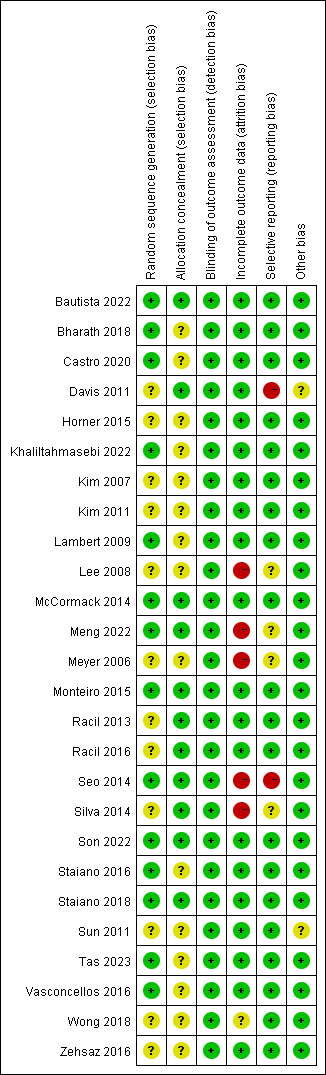

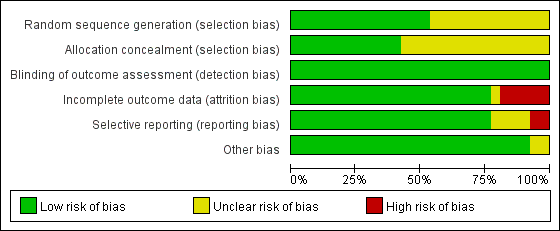


**Table S4**. Quality assessment (GRADE for exercise intervention versus control)

| Exercise compared to no exercise for glycolipid metabolism | | | |
| --- | --- | --- | --- |
|  | | | |
| Outcomes | № of participants  (studies) | Certainty of the evidence (GRADE) | Relative effect (95% CI) |
| FBG | 742 (18 RCTs) | ⨁⨁◯◯ LOW ^a,b^ | SMD-0.42  [-0.73, -0.12] |
| FINS | 780 (19 RCTs) | ⨁⨁◯◯ LOW ^a,b^ | SMD-0.81  [-1.13, -0.49] |
| TC | 773 (22 RCTs) | ⨁⨁⨁◯ MODERATE ^a^ | SMD -0.18  [-0.34, -0.01] |
| TG | 785 (22 RCTs) | ⨁⨁⨁◯ MODERATE ^a^ | SMD -0.40  [-0.56, -0.25] |
| LDL-c | 785 (22 RCTs) | ⨁⨁⨁◯ MODERATE ^a^ | SMD -0.28  [-0.44, -0.12] |
| HDL-c | 785 (22 RCTs) | ⨁⨁⨁◯ MODERATE ^a^ | SMD 0.26  [0.11, 0.40] |
| Abbreviations: FBG, fasting blood glucose; FINS, fasting insulin; TC, Total cholesterol; TG, Triglycerides; HDL, high-density lipoprotein; LDL, low-density lipoprotein; CI: Confidence interval; SMD: Standardized mean difference. | | | |
| GRADE Working Group grades of evidence High certainty: We are very confident that the true effect lies close to that of the estimate of the effect Moderate certainty: We are moderately confident in the effect estimate: The true effect is likely to be close to the estimate of the effect, but there is a possibility that it is substantially different Low certainty: Our confidence in the effect estimate is limited: The true effect may be substantially different from the estimate of the effect Very low certainty: We have very little confidence in the effect estimate: The true effect is likely to be substantially different from the estimate of effect | | | |

Explanations

a. Less than 75% of studies had a low risk of bias.

b. Heterogeneity: I2 >50%

**
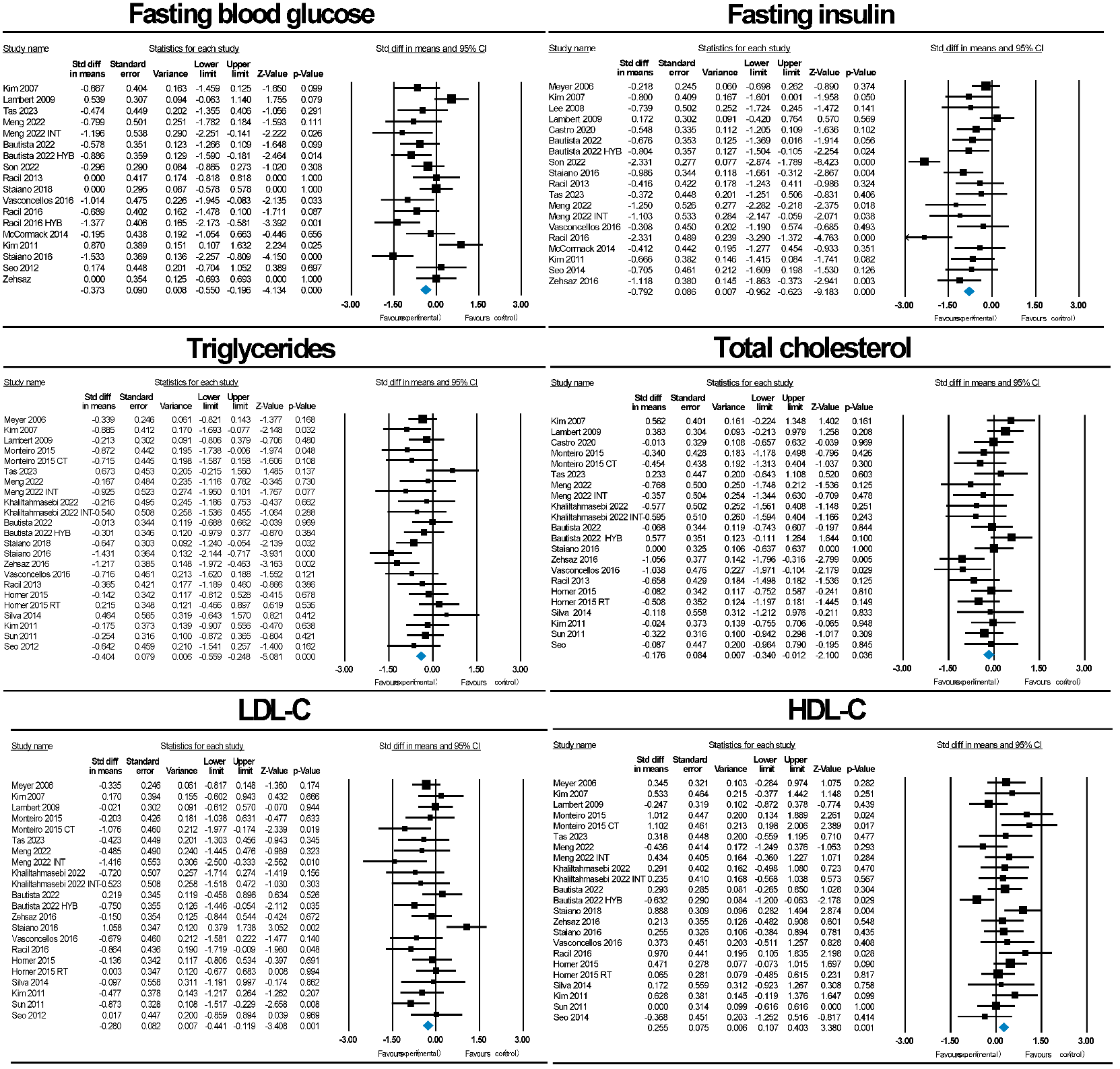
Figure S2.** Forest plots of eligible comparisons of Fasting blood glucose (FBG), fasting insulin (FINS), total cholesterol (TC), triglycerides (TG), low-density lipoprotein (LDL-C) and high-density lipoprotein (HDL-C).

**Figure S3** Funnel plots graphics of fasting blood glucose (FBG), fasting insulin (FINS), total cholesterol (TC), triglycerides (TG), low-density lipoprotein (LDL-C) and high-density lipoprotein (HDL-C).


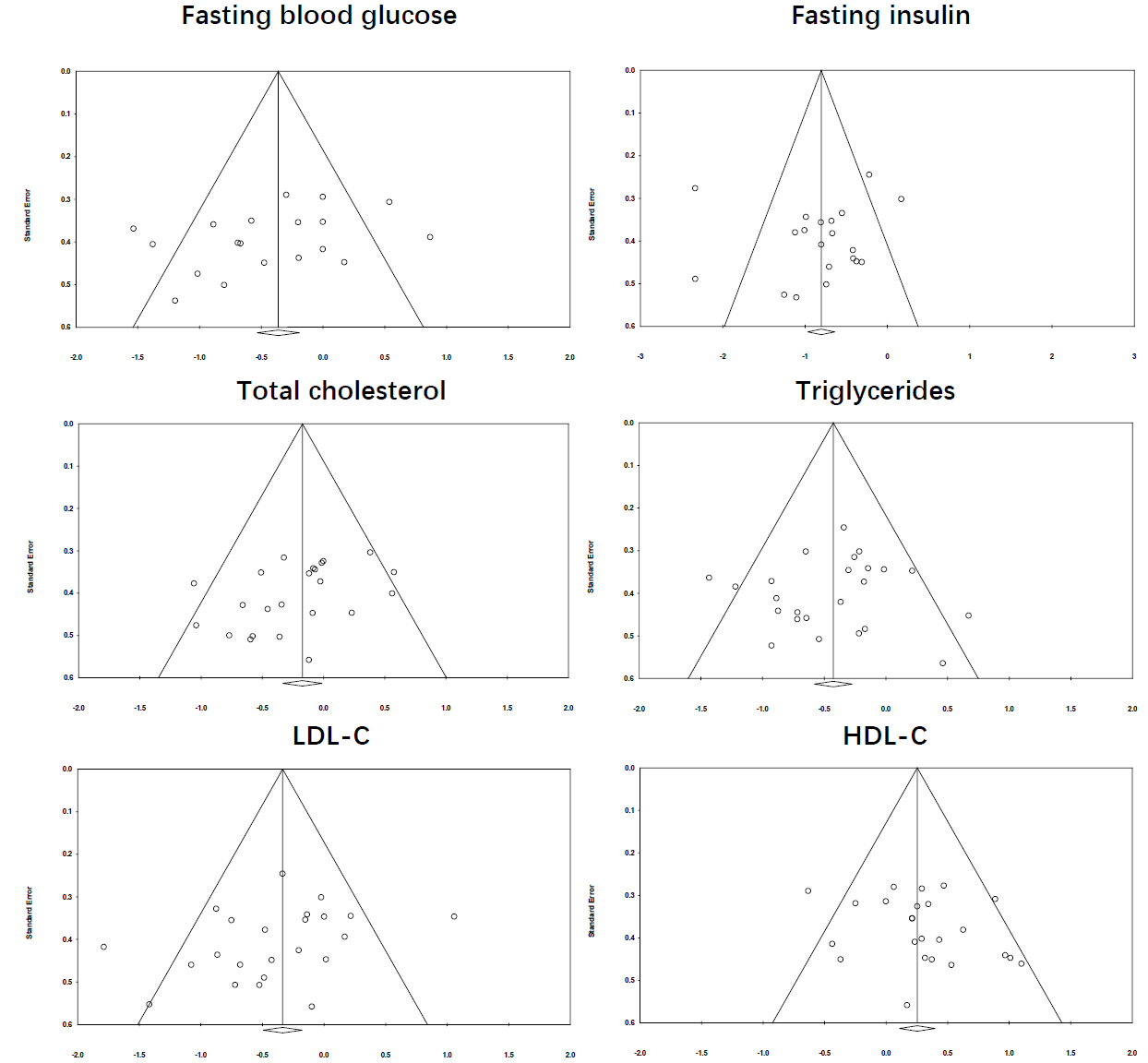


**Table S5.** Egger’s test of eligible comparisons of Fasting blood glucose (FBG), fasting insulin (FINS), total cholesterol (TC), triglycerides (TG), low-density lipoprotein (LDL-C) and high-density lipoprotein (HDL-C).

| **Outcomes** | **Egger’s intercept** | **SE** | **95% CI** | **p** |
| --- | --- | --- | --- | --- |
| FBG | -3.54 | 2.18 | [-8.13，1.06] | 0.12 |
| FINS | -0.71 | 1.96 | [-4.84，3.40] | 0.71 |
| TC | -2.70 | 1.32 | [-5.46, 0.04] | 0.05 |
| TG | -0.31 | 1.25 | [-2.90, 2.27] | 0.80 |
| LDL-C | -3.90 | 1.92 | [-7.90, 0.09] | 0.06 |
| HDL-C | 1.65 | 1.27 | [-1.00, 4.30] | 0.20 |

**Appendix S1.** List of included studies.

1. Meyer, A.A., et al., *Improvement of Early Vascular Changes and Cardiovascular Risk Factors in Obese Children After a Six-Month Exercise Program.* Journal of the American College of Cardiology, 2006. **48**(9): p. 1865-1870.

2. Kim, E.S., et al., *Improved insulin sensitivity and adiponectin level after exercise training in obese Korean youth.* Obesity, 2007. **15**(12): p. 3023-3030.

3. Kim, H.J., et al., *Effects of exercise-induced weight loss on acylated and unacylated ghrelin in overweight children.* Clinical endocrinology, 2008. **68**(3): p. 416‐422.

4. Farpour-Lambert, N.J., et al., *Physical Activity Reduces Systemic Blood Pressure and Improves Early Markers of Atherosclerosis in Pre-Pubertal Obese Children.* Journal of the American College of Cardiology, 2009. **54**(25): p. 2396-2406.

5. Seo, D.Y., et al., *Yoga training improves metabolic parameters in obese boys.* The Korean journal of physiology & pharmacology, 2012. **16**(3): p. 175‐180.

6. Davis, J.N., et al., *Startup circuit training program reduces metabolic risk in Latino adolescents.* Medicine and science in sports and exercise, 2011. **43**(11): p. 2195‐2203.

7. Duft, R.G., et al., *Altered metabolomic profiling of overweight and obese adolescents after combined training is associated with reduced insulin resistance.* Scientific reports, 2020. **10**(1): p. 16880.

8. Monteiro, P.A., et al., *Concurrent and aerobic exercise training promote similar benefits in body composition and metabolic profiles in obese adolescents.* Lipids in Health and Disease, 2015. **14**(1).

9. Tas, E., et al., *Effects of short-term supervised exercise training on liver fat in adolescents with obesity: a randomized controlled trial.* Obesity, 2023. **31**(11): p. 2740-2749.

10. Meng, C., et al., *Effects of school-based high-intensity interval training on body composition, cardiorespiratory fitness and cardiometabolic markers in adolescent boys with obesity: a randomized controlled trial.* BMC Pediatrics, 2022. **22**(1).

11. Khaliltahmasebi, R., V. Minasian, and S. Hovsepian, *Effects of two different school-based training on serum miR15b expression and lipid profile of adolescents with obesity.* International Journal of Preventive Medicine, 2022. **13**(1).

12. González-Ruíz, K., et al., *Exercise dose on hepatic fat and cardiovascular health in adolescents with excess of adiposity.* Pediatric Obesity, 2022. **17**(4).

13. Kim, J., et al., *The effects of a 12-week jump rope exercise program on body composition, insulin sensitivity, and academic self-efficacy in obese adolescent girls.* Journal of Pediatric Endocrinology and Metabolism, 2020. **33**(1): p. 129-137.

14. Bharath, L.P., et al., *Combined resistance and aerobic exercise training reduces insulin resistance and central adiposity in adolescent girls who are obese: randomized clinical trial.* European journal of applied physiology, 2018. **118**: p. 1653-1660.

15. Wong, A., et al., *The effects of a 12-week combined exercise training program on arterial stiffness, vasoactive substances, inflammatory markers, metabolic profile, and body composition in obese adolescent girls.* Pediatric Exercise Science, 2018. **30**(4): p. 480-486.

16. Staiano, A.E., et al., *Home-based exergaming among children with overweight and obesity: a randomized clinical trial.* Pediatric obesity, 2018. **13**(11): p. 724‐733.

17. Zehsaz, F., N. Farhangi, and M. Ghahramani, *Exercise training lowers serum chemerin concentration in obese children.* Science & sports, 2017. **32**(1): p. 39‐45.

18. Staiano, A.E., et al., *A randomized controlled trial of dance exergaming for exercise training in overweight and obese adolescent girls.* Pediatric Obesity, 2017. **12**(2): p. 120-128.

19. Vasconcellos, F., et al., *Health markers in obese adolescents improved by a 12-week recreational soccer program: a randomised controlled trial.* Journal of sports sciences, 2016. **34**(6): p. 564-575.

20. Racil, G., et al., *Plyometric exercise combined with high-intensity interval training improves metabolic abnormalities in young obese females more so than interval training alone.* Applied physiology, nutrition, and metabolism = Physiologie appliquee, nutrition et metabolisme, 2016. **41**(1): p. 103-109.

21. Racil, G., et al., *Effects of high vs. Moderate exercise intensity during interval training on lipids and adiponectin levels in obese young females.* European Journal of Applied Physiology, 2013. **113**(10): p. 2531-2540.

22. Horner, K., et al., *Effect of Aerobic versus Resistance Exercise on Pulse Wave Velocity, Intima Media Thickness and Left Ventricular Mass in Obese Adolescents.* Pediatric exercise science, 2015. **27**(4): p. 494‐502.

23. Silva, D.A.S., E.L. Petroski, and A. Pelegrini, *Effects of aerobic exercise on the body composition and lipid profile of overweight adolescents.* Revista Brasileira de Ciências do Esporte, 2014. **36**(2): p. 295-309.

24. McCormack, S.E., et al., *Effects of exercise and lifestyle modification on fitness, insulin resistance, skeletal muscle oxidative phosphorylation and intramyocellular lipid content in obese children and adolescents.* Pediatric Obesity, 2014. **9**(4): p. 281-291.

25. Kim, J.Y., et al., *Improved Insulin Resistance, Adiponectin and Liver Enzymes without Change in Plasma Vaspin Level after 12 Weeks of Exercise Training among Obese Male Adolescents.* The korean journal of obesity, 2011. **20**(3): p. 138‐146.

26. Sun, M.X., et al., *One-hour after-school exercise ameliorates central adiposity and lipids in overweight Chinese adolescents: a randomized controlled trial.* Chin Med J (Engl), 2011. **124**(3): p. 323-9.
